# Immunological Aspects Involved in the Degeneration of Cryopreserved Arterial Allografts

**DOI:** 10.3389/fsurg.2020.616654

**Published:** 2020-12-22

**Authors:** Mario González-Gay, Rocío López-Martínez, Sara Busto-Suárez, Mariel Estefanía Riedemann-Wistuba, María Ángeles Menéndez-Herrero, Francisco Álvarez-Marcos, Manuel Alonso-Pérez, Rebeca Alonso-Arias

**Affiliations:** ^1^Department of Angiology and Vascular Surgery, Central University Hospital of Asturias, Oviedo, Spain; ^2^Department of Immunology, Central University Hospital of Asturias, Oviedo, Spain

**Keywords:** cryopreserved arteries, vascular transplant, allograft, homograft, aneurysm, chronic rejection, critical ischemia, vascular infection

## Abstract

**Introduction:** Cryopreserved arterial allografts have remained an option in patients requiring distal revascularization or associated with vascular infection, in the absence of a valid autogenous saphenous vein. The objective of this study is to describe the different clinical, anatomopathological, and immunological findings related to vascular transplant rejection.

**Methods:** In a prospective trial, 35 patients who underwent cryopreserved allogeneic arterial bypass were studied, including demographics and conduit patency. Anti-HLA antibody production was stablished prior to the surgery, 7 days, 1, 3 months, and every 3 months since. Clinical and ultrasound evaluation was added after the first month. Donor HLA-typing was retrieved whenever available, allowing for the characterization and quantification of donor specific antibodies. Cytotoxic crossmatch test was also performed. A second group of patients with allograft degenerations registered during the follow up period was studied. In this group, exclusively for aneurysm description and histopathological analysis, they were included those degenerated vascular transplants from the original series, but also those implanted prior to the beginning of the study and degraded during follow up.

**Results:** All patients studied displayed an increase in anti-HLA antibodies one month after the intervention, regarding bypass patency. In total, 14 patients fulfilled requirements for the study of donor specific antibodies, equally showing IgG production detectable one month after surgery. The presence of complement-fixing antibodies was also confirmed. Antibody levels were not related to graft degeneration. No specific immune markers able to predict aneurysmal development and evolution were found. From the original group, 3 patients suffered aneurysmal degeneration during follow up, together with 9 bypasses previously implanted. Average time until the first degeneration was 33 ± 19.7 months, with 30.6 ± 17.7 and 54.5 ± 2.5 months for a second and third degeneration, when occurring. Therefore, subsequent vascular transplants frequently augmented the time for new degenerations, despite increasing sensibilization. Samples from eight degenerated allografts were available for analysis, unexpectedly showing inflammatory infiltrate in only four cases and immune complex deposition in 7.

**Conclusions:** Immune response against vascular transplants was confirmed in all cases, but chronic rejection did not necessarily provoke bypass degradation or reduced the time for new aneurysms to develop in subsequent allografts.

## Introduction

The discovery of synthetic vascular grafts, like polytetrafluoroethylene and Dacron, expanded the possibilities of revascularization with a new generation of readily available synthetic vascular grafts. Nevertheless, the autogenous great saphenous vein (GSV) has remained the gold standard in the setting of local infection and when an infrageniculate bypass is required for limb salvage, submitting the best performance in terms of resistance to reinfection, patency and limb salvage (1).

However, 20–45% of the patients present a non-viable or absent ipsilateral GSV, owing to venous insufficiency, varicose veins surgery or previous use as revascularization graft (2, 3). Furthermore, when a new bypass is needed for a second revascularization, the lack of a valid GSV can reach at least 50% of the cases (4). For those reasons, imaginative solutions have aroused and cryopreserved vascular transplants have found their place in the surgical arsenal. The use of umbilical-cord vein allografts (5) or bovine-origin xenografts (6) has tried to address the same issues, although their implementation in daily clinical practice has been mostly marginal.

This cryopreserved vascular grafts offered acceptable performance in demanding scenarios, but not without a cost, both in terms of logistics and complications. The difficulty to harvest, process and preserve the vessels increases the price and the resources needed to maintain a suitable tissue bank. Consequently, not every center can meet these requirements. Cryopreserved allografts would need to be obtained then from external public or private banks. Moreover, vascular transplantation had to face major setbacks since its beginnings in the form of vessel degeneration and aneurysm formation (7). The use of fresh allografts was slowly abandoned in favor of cryopreservation in part for this reason, with just a few groups still working with tissue in cold ischemia (8, 9).

The process of cryogenization was supposed to reduce this complication, increasing the accessibility of these conduits. It was no longer required implantation a few hours or days after extraction, allowing storage for years with prompt availability. However, even after the important progress achieved with freezing and thawing protocols using dimethyl sulfoxide (DMSO), a small number of allografts would still suffer aneurysmal degradation. This complication has been the source of major concern and study, resulting in controversial conclusions regarding patency, antigenicity and immune response.

The objective of this research is to describe the different clinical, anatomopathological and immunological findings related to cryopreserved vascular transplant rejection, ultimately leading to aneurysmal degeneration. To achieve this, we analyzed our series of cryopreserved arterial bypasses and the aneurysms observed during the study period.

## Materials and Methods

The first study group consisted of a prospective observational cohort of patients who underwent revascularization by means of cryopreserved arterial allografts at the Department of Vascular Surgery of Asturias Central University Hospital (HUCA), Oviedo, Spain, from November 2015 to May 2018. Follow-up period covered until December 2019. The indication for revascularization included both prosthetic or native artery infection and critical limb ischemia (stages 4–6 in the Rutherford Classification), in the absence of valid autologous GSV or lesser saphenous vein. Allografts implanted in relation with an initial infectious indication were also included, since the process should not alter the immunological results that are key to determine allograft rejection in our study. We do not use the arm veins as an alternative in these patients because their benefit is a subject of controversy, offering poor results with reintervention rates that can reach 83%, especially when splicing with 2 or more anastomoses is required (10–12). In the study we excluded patients who previously underwent solid organ transplantation and/or those under immunosuppressive treatment, as this could alter the immunological response after the vascular transplant. In case the length of the bypass required the anastomoses of two different arteries, both belonged to the same donor, except if otherwise specified. The grafts were obtained from Asturias Blood and Tissue Community Bank or delivered from Burgos University Hospital and Catalonian Blood and Tissue Bank, whenever a cryopreserved artery was not locally available. All vessels were harvested from multi-organ donors under the National Transplant Program, extracting the ilio-tibioperoneal trunk segment within 6 h after exitus (warm ischemia time). Processing and freezing took place in <8 h since extraction, keeping the tissue refrigerated at all times at 4°C in Dulbecco's Modified Eagle Medium (DMEM), with an antibiotic combination of Clindamycin, Amikacin and Vancomycin (cold ischemia time). A small sample was previously taken for bacteriological control.

Cryopreservation took place in 180 ml of DMEM and 20 ml of DMSO, reducing the temperature in the programmable freezer from +4 to −80°C, at a lineal rate of −1°C per minute. Once the thermal decrease had been reached, the artery was stored at −140°C in the vapor phase of a liquid nitrogen tank. Thawing was performed in 37°C saline until the tissue was completely unfrozen, rinsing DMSO three times with saline for at least 2 min each time. Implantation was carried out immediately after thawing. Owing to regular use and obvious supply restrictions, cryopreserved arteries at our center are stored for less than a year before grafting.

Based on our protocols and current scientific evidence (13–15), no ABO or Rhesus compatibility was studied prior to graft implantation. On the other hand, donor/recipient Human Leucocyte Antigen (HLA) compatibility could not be purposely achieved in most cases due to stock limitations and time requirements in emergency interventions.

Demographic information, comorbidities, surgical indication, and follow up data were collected. Clinical evaluation was conducted 1 and 3 months after surgery, with periodical reviews every 3 months thereafter. Bypasses were examined with ultrasound imaging, registering patency, diameter measurements at regular points in the length of the artery, as well as the presence of graft stenosis, if it were the case. Additional computed angiotomography was requested when required by patient's evolution.

Primary, primary assisted and secondary patency rates, limb salvage (defined as absence of major amputation, excluding transmetatarsal) and patient survival were calculated using the Kaplan-Meier method. Statistical analysis was performed using SPSS Statistics version 20 (SPSS Inc., Chicago, IL, USA).

All patients were subject to anti-HLA antibody analysis from serum obtained before the intervention, as well as 1 week, 1 month, and every 3 months since. This was performed until completing 1 year, bypass thrombosis or graft explantation without revascularization with a new cryopreserved artery. The presence of immunoglobulin G (IgG) antibodies against HLA antigens was screened by using Lifecodes LSA Class I and Class II single antigen bead assays kits (Immucor Inc.), with immunoassays analyzed by using a Luminex 200 (Luminex Corp., Austin, TX, USA).

Transplant recipients with at least 1-month allograft patency underwent HLA typing through DNA amplification by Polymerase Chain Reaction using Lifecodes HLA sequence-specific oligonucleotides Typing Kits (Immucor Inc., Waukesha, WI, USA). Samples were measured in the flow cytometer Luminex 2000. HLA typing was obtained in the same way for donors whose vascular tissue was harvested and preserved at Asturias Blood and Tissue Community Bank. This profile could not be acquired for cryopreserved arteries delivered from other national centers. Donor specific antibodies (DSA) were registered in terms of time from surgery and median fluorescence intensity (MFI).

Complement-Dependent Cytotoxicity (CDC) crossmatch test was also performed aiming to detect anti- HLA antibody ability to fixing complement. Peripheral blood mononuclear cells (PBMC) from random bone marrow donors were isolated from peripheral blood anticoagulated with ethylenediaminetetraacetic acid (EDTA) by centrifugation on Ficoll-Hypaque gradients (Lymphoprep; Nycomed, Oslo, Norway). PBMC were resuspended in RPMI 1640 (BioWhitaker, Verviers, Belgium) at 2.5 × 10^6^ cells /mL and mixed (1 mL) in a Terasaki plate with 1 mL of serum from each patient. Serum and cells were incubated 30 min at room temperature and after complement addition 60 min at room temperature. Then Fluoroquench (One Lambda) was dispensed in the microcytotoxicity assay, staining both live (green) and dead (red) cells which can then be distinguished by fluorescence microscopy. The test was considered positive when donor-specific antibodies bound to lymphocytes, activating complement and causing cell lysis in at least 20% of the cells in each well.

A second group of patients with allograft degenerations registered during the follow up period was studied. In this group, exclusively for aneurysm description and histopathological analysis, they were included those degenerated vascular transplants from the original series, but also those aneurysms detected during our inclusion period, although implanted prior to the beginning of the study. This group was created to reduce the follow up time required to register a sufficient number of degenerated bypasses. Whenever it was possible to obtain an allograft's sample due to new revascularization surgery, immunohistochemistry and immunofluorescence were analyzed as long as the bypass was patent. Additionally, control samples from native artery aneurysms belonging to random patients were also studied. Evaluation of IgG, C3, C4 and HLA class I was performed using direct immunofluorescence. Immunohistochemical analysis was also completed for IgG, C3, C4 and HLA class I, including staining for actin and elastic fibers. No anatomopathological sample was affected by local infection, as that could alter the results of the immunohistochemistry and immunofluorescence.

Approval for this investigation was obtained from our center's Ethics Committee, and the protocol was in accordance with the Declaration of Helsinki for medical research involving human subjects (16).

## Results

In total, 35 patients who underwent revascularization surgery with cryopreserved arteries during the inclusion period were included in the study. The mean age was 72.8 years (range 46–100). Twenty-eight patients (80%) were male. Surgical indications were native or prosthetic infection in 10 cases (28.5%) and limb threatening ischemia in 25 patients (71.4%). Additionally, 8 out of the 10 patients with vascular infection also had a previous history of chronic limb ischemia. Regarding ischemic indication, 52% suffered from rest pain, and the remaining 48% were associated with tissue loss or gangrene. Moreover, 82.8% of all the patients (*n* = 29) had undergone previous revascularization surgery at the time of the vascular transplant. Table 1 shows the parameters previously described, including risk factors and comorbidities.

**Table 1:** Patient characteristics.

**Demographic data**	
Mean age, years ± SD (range)	72.8 ± 12.3 (46–100)
Sex, male/female (%)	28/7 (80/20)
**Risk factors and comorbidities**	
Smoking/ Ex (%)	9/14 (25.7/40)
Hypertension (%)	25 (71.4)
Diabetes (%)	22 (62.8)
Dyslipidaemia (%)	15 (42.8)
COPD (%)	8 (22.8)
Chronic renal failure (%)	10 (28.5)
Ischemic cardiopathy (%)	8 (22.8)
Other cardiac diseases (%)	10 (28.5)
TIA or stroke (%)	4 (11.4)
Chronic limb ischemia (%)	32 (91.4)
Neoplasia (%)	6 (17.1)
**Surgical indication**	
Limb threatening ischemia (%)	25 (71.4)
Rest pain (%)	13 (52)
Tissue loss (%)	12 (48)
Infection (%)	10 (28.6)
Previous revascularization (%)	29 (82.8)

*SD, Standard Deviation; COPD, Chronic Obstructive Pulmonary Disease; Chronic renal failure, glomerular filtration rate <60 mL/min; Other cardiac diseases, valvopathy, cardiac failure, or atrial fibrillation; TIA, Transient Ischemic Attack*.

Most cryopreserved allografts (77.1%, *n* = 27) had an infrageniculate artery as the distal target vessel, with 15 bypasses (42.8%) anastomosed to a distal trunk. Suprainguinal bypasses (*n* = 2), exclusively involving the aorta and the iliac arteries, as well as all suprageniculate grafts, were indicated because of infection (Table 2).

**Table 2:** Allograft location regarding distal target vessel.

**Suprainguinal**	
Aorta to iliac	2
**Suprageniculate**	
Iliac to femoral	5
Femoral to popliteal (above-knee)	1
**Infrageniculate**	
Iliac to TP trunk, to posterior tibial	1
Iliac to anterior tibial	1
Femoral to popliteal (below-knee)	12
Femoral to anterior tibial	3
Femoral to posterior tibial	5
Femoral to peroneal	3
Femoral to TP trunk	1
Popliteal (above-knee) to posterior tibial	1

*TP trunk, Tibioperoneal trunk*.

Only three patients were lost to follow-up for other reasons than death, bypass thrombosis or allograft replacement. Median follow-up was 27.6 months, with a range between 9 and 1,340 days (interquartile range or IQR of 16.5–35.5 months). Fifteen patients needed reintervention due to complications related to the cryopreserved artery (42.8%), with nine cases requiring more than one procedure, for a total of 29 secondary procedures. Six interventions involved bypasses at risk of failure, 12 were performed in order to rescue already thrombosed grafts, and 11 cryopreserved arteries were replaced for new bypasses. Two patients required emergency surgery to control active bleeding, both in relation with current infection.

**Figure 1:**
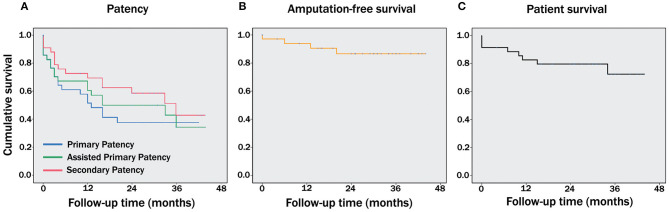
Kaplan-Meier survival functions showing long term outcomes. Cumulative survival curves for bypass patency, including primary, primary assisted and secondary patencies **(A)**, amputation-free survival **(B)**, and patient survival **(C)**.

Primary patency at 1, 2, and 3 years was 58, 38, and 38%, respectively, with a median of 12 months (IQR = 2–27). Assisted primary patency was 67, 49, and 44% at 1, 2, and 3 years (the median was 13 months and the IQR = 2–31). Finally, secondary patency for the same intervals was 73, 62, and 52%, respectively. The median secondary patency was 16 months, and the IQR = 3–32 (Figure 1A). Because of patients lost to follow up, assisted primary patency curve drops below primary patency curve after 39 months of the vascular transplant. Only four patients (11.4%) required major amputation, with a limb salvage rate of 94% at 1 year and 87% at 2 and 3 years (Figure 1B). Regarding patient survival, eight patients (22.8%) died during follow-up, with three deaths related to their ischemic/infection condition, at 9, 16, and 22 days (early mortality). No deaths were associated with allograft degeneration. Patient survival at 1, 2, and 3 years was 83, 79, and 74%, respectively (Figure 1C).

During follow-up, three out of the 35 patients who initially entered the study registered aneurysmal degeneration 7, 22, and 28 months after surgery (8.5% of our series). Two cases underwent bypass replacement, and the other one was scheduled for regular monitoring with ultrasound scans due to its small stable diameter. Given sample size, statistical power was too low to establish correlations between population demographics and bypass degeneration with statistical significance.

Another nine patients who had a vascular transplant implanted prior to the beginning of this study, registered allograft degeneration during the inclusion period. Altogether, we studied 12 allograft degeneration, with a mean age of 64.5 years (range 48–84) and none of them related to previous infection. Ten bypasses were located on distal trunks and the other two targeted the popliteal artery below the knee (16.7%). For six patients this was the second degeneration on different cryopreserved arteries, while two patients registered their third event. Average evolution time until the first degeneration was 33 ± 19.7 months (range 7–71), 30.6 ± 17.7 months for a second degeneration (range 14–68) and 54.5 ± 2.5 months for a third episode (52 and 57 months). Both patients with aneurysmal degradation of three different bypasses, and one affecting two separate grafts, had in common a previous history of native arterial aneurysms in different locations. This accounts for half the patients with multiple episodes of degeneration (Table 3).

**Table 3:** Allograft degenerations.

**Patient**	**Age**	**BP type**	**Degen.**	**Time 1st Degen.**	**Time 2nd Degen.**	**Time 3rd Degen.**
1	66	B-K Pop.	1	28	–	–
2	84	Distal	1	22	–	–
3	62	Distal	2	7	14	–
4	67	Distal	2	27	17	–
5	64	Distal	3	14	27	52
6	71	Distal	2	23	32	–
7	48	Distal	2	56	68	–
8	60	Distal	3	67	26	57
9	55	B-K Pop.	1	42	–	–
10	75	Distal	1	23	–	–
11	58	Distal	1	19	–	–
12	64	Distal	1	71	–	–

*Age, age in years at the time of the first bypass; BP type, Bypass type regarding distal target vessel; B-K Pop., Bellow-knee popliteal artery; Degen., Number of degenerations affecting different allografts; Time Degen., Time in months from the surgery until allograft degeneration*.

In total, eight samples from degenerated cryopreserved arteries were available for analysis by the Pathology Department, while four cases were treated in a way that did not make possible that study. In addition, two control samples from random patients presenting native artery aneurysms (popliteal and humeral) were retrieved for comparison.

**Figure 2:**
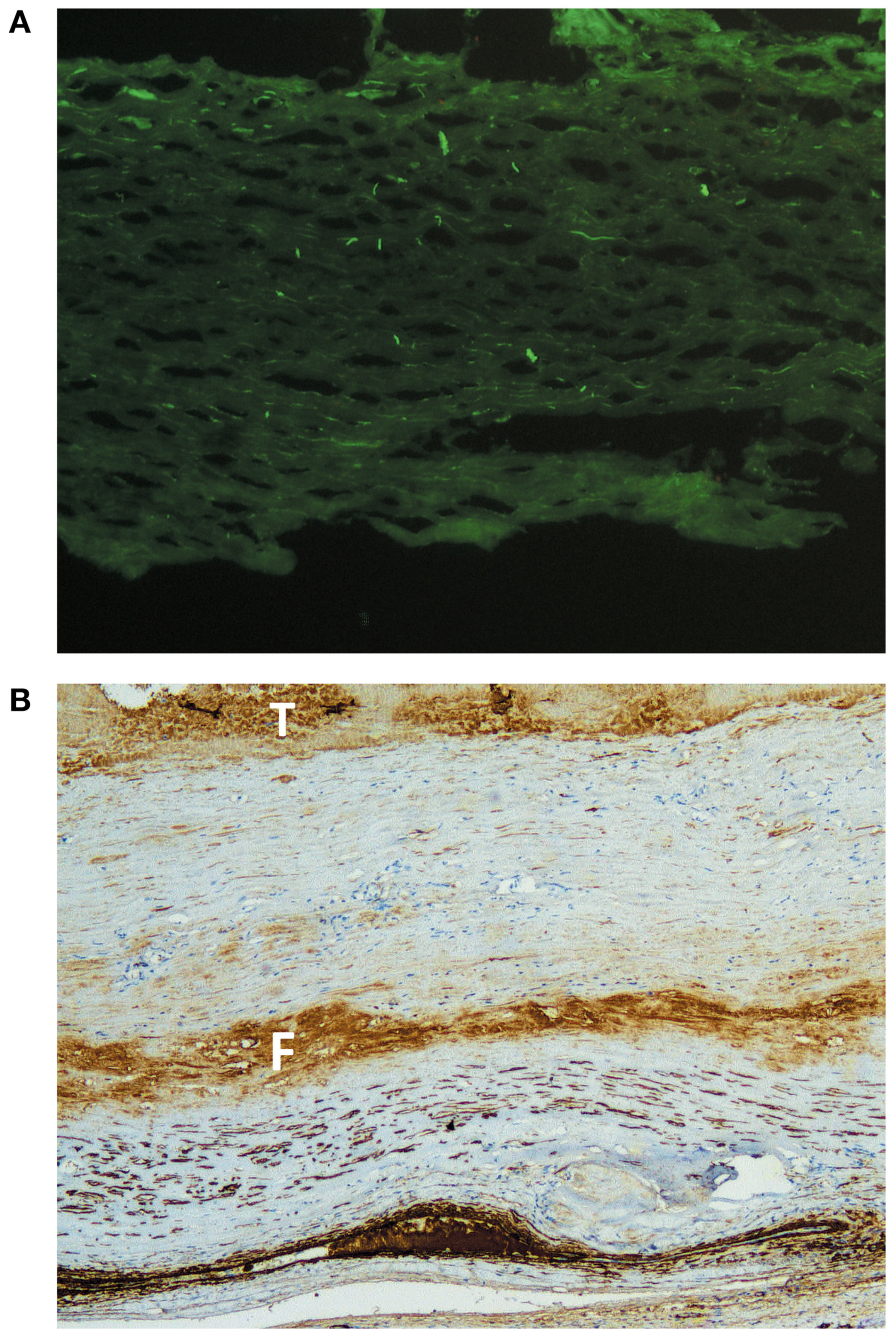
Immunohistological analysis of control native artery aneurysms. **(A)** Immunofluorescence staining for IgG in a native humeral artery aneurysm. Homogenous distribution, suggesting the absence of immune complex deposition. Original magnification ×20. **(B)** Immunohistochemical staining for actin in a native popliteal artery aneurysm. The image shows a highly degenerated arterial wall in which the muscular layer has been mostly substituted by fibrous tissue (the F highlights a fibrin band). Intraluminal thrombus is marked with a T, and the darker layer at the bottom corresponds to an artifact. There is no evidence of immune complexes. Original magnification ×4.

Control aneurysms showed chronic inflammation affecting all layers and degenerative parietal changes. Intima and media cellularity were dependant on the level of degeneration/fibrosis, ranging from alteration in layer distribution to complete loss of the tunica muscularis in the area affected. Both immunohistochemistry and immunofluorescence were negative for significant immune deposits (Figure 2).

**Figure 3:**
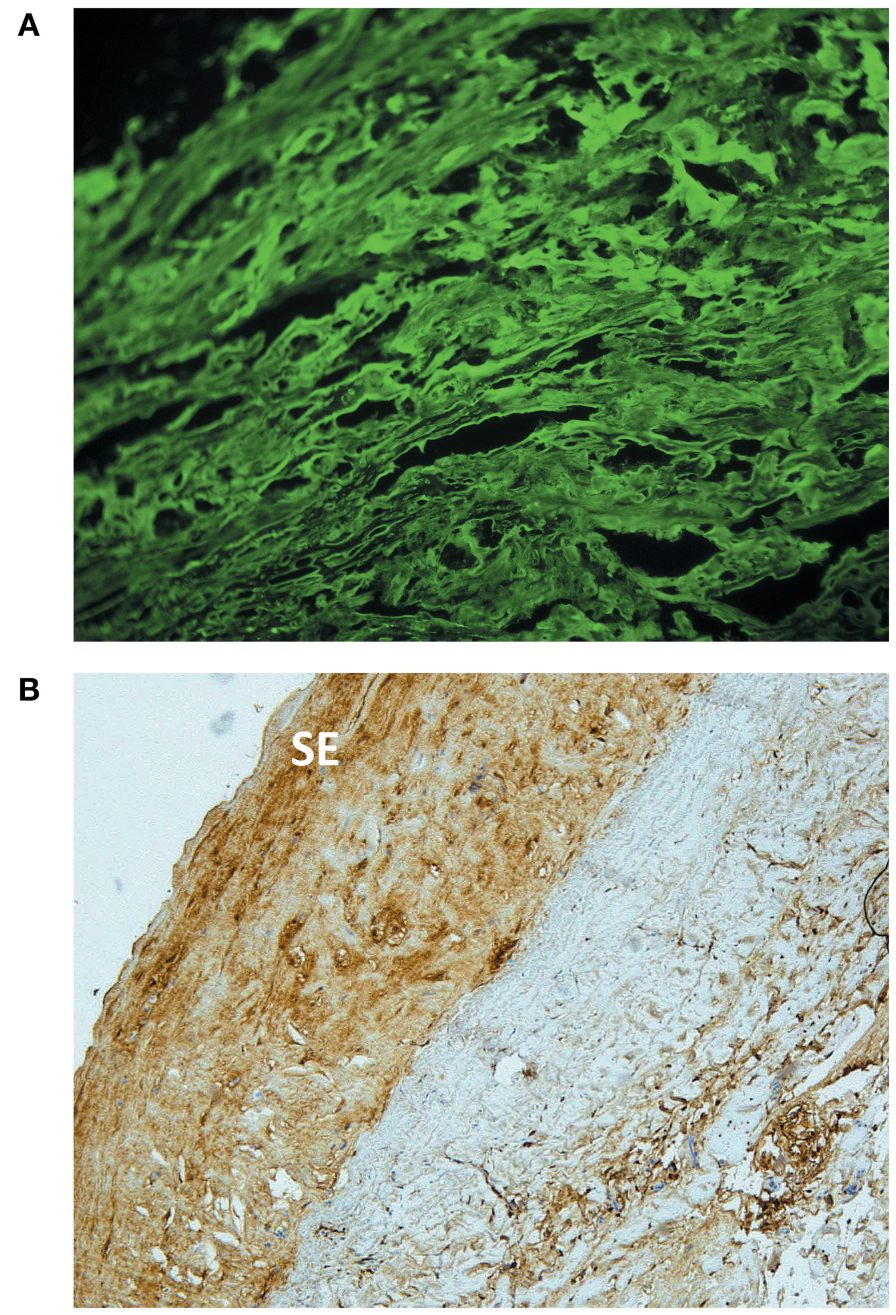
Immunohistological analysis of a degenerated cryopreserved artery allograft. **(A)** Immunofluorescence staining of a degenerated cryopreserved artery excised 24 months after transplantation. The sample shows interstitial immune complex deposition, positive for IgG. Original magnification ×20. **(B)** Immunohistochemical analysis of the same allograft. IgG deposits located predominantly in the neointima, with a higher density in the subendothelial area (SE). Original magnification ×10.

On the other hand, degenerated cryopreserved arteries showed hypocellularity of all layers, particularly affecting the tunica media. This loss could be almost complete on occasions, although always with a few viable cells remaining. A fibrosing reparative process of the arterial wall could be observed in the allografts, with the presence of active fibroblasts and fragmentation of elastic fibers. The intima had been progressively substituted by granulation tissue. Nevertheless, the presence of chronic inflammation, with infiltration of mononuclear cells, was absent or minimal in at least 50% of our samples. Three aneurysms also showed areas of calcification and sclerosis, displaying foamy histiocytes with intracellular cholesterol that could be observed in all the layers. This diffuse distribution does not correspond to that of the classic atheromatous plaque. Finally, there was a significant presence of immune complex deposition in the tunica intima and media, positive for C3, IgG and HLA class I, and mainly affecting the most degenerated and acellular areas (Figure 3). These findings were absent in one allograft, and marginal in another one.

All patients showed a detectable immunological reaction after transplantation, consisting in a significant increase of anti-HLA antibodies in peripheral blood 1 month after surgery. These changes were not observed in the first control, 1 week after the vascular transplant, but were consistent thereafter. Although anti-HLA antibodies were mainly produced against donor HLA, due to shared epitopes between alleles they were also detected against other specificities.

Serial DSA were evaluated in 14 patients with 16 allografts, in which donor HLA profile was available and with a minimum bypass patency of 1 month. This allows comparison between donor and receptor, establishing specific antibody generation in time. Therefore, early thrombosed bypasses and allografts obtained from other centers were excluded. We registered no random HLA-matched allografts in our series, except for some alleles or genes. The study detected significant production of IgG antibodies specific against donor histocompatibility antigens, although this formation was significantly higher against HLA-A, HLA-B, and HLA-DR antigens. This was also the case for antibodies produced in relation to HLA-mismatching. DSA were seen for HLA-A, HLA-B, and HLA-C in 92.8, 92.8, and 28.5% of the receptors, respectively, while for HLA-DR, HLA-DQ, and HLA-DP mismatches, those percentages correspond to 71.4, 57.1, and 35.7%.

After the vascular transplant, 13 patients (92.8%) were considered hypersensitized, displaying a Panel Reactive Antibody (PRA) over 95%. Only one patient had a lower PRA of 76.7%. If new allografts were implanted, the PRA value increased accordingly, rendering the receptor even more immunized. Table 4 registers HLA profiles of both donors and recipients, highlighting antigens against which detected DSA were formed. Serological typing is shown, except in alleles with no serological equivalent, in which case the first digits are chosen. In the case of 14A and 14B, both allografts were implanted into the same recipient very proximate in time (less than a week), with no antibody analysis between both procedures. Patients 2 and 10, 5 and 6, and 1 and 13 received cryopreserved arteries from the same donors, respectively. Aneurysmal degeneration was observed during follow up in patients 6 and 12.

**Table 4:** HLA phenotypes and DSA production.

**ID**	**HLA-class I (Patient)**	**HLA-class II (Patient)**	**HLA-class I (Donor)**	**HLA-class II (Donor)**
1	A1, 68; B44, 53; C4, 16	DR7, 1; DQ2, 5; DP11, 17	**A**1, **2**; **B**44, **57**; C5, 7	**DR**7, **12**; **DQ7**, **9**; DP4, 6
2	A2, 29; B18, 44; C4, 7	DR7, 11; DQ2, 7; DP4, –	**A**2, **3**; **B35**, **50**; C4, 6	**DR**7, **17**; DQ2, –; DP4, 7
3	A2, –; B51, 58; C7, 15	DR13, 17; DQ2, 6; DP4, 13	**A1**, –; **B8**, **57**; C6, 7	**DR7**, 17; DQ2, –; **DP2**, 4
4	A3, 11; B7, 18; C5, 7	DR15, 17; DQ2, 6; DP2, 4	**A2**, 3; **B8**, **44**; C5, 7	**DR11**, 13; **DQ**6, **7**; **DP**4, **17**
5	A11, 30; B7, 44; C4, 7	DR4, –; DQ4, 8; DP3, 4	**A2**, **68**; B45, **51**; C6, 15	**DR13**, **14**; **DQ7**, 8; DP3, 10
6	A29, 30; B8, 38; C7, 12	DR11, 17; DQ2, 7; DP4, 11	**A2**, **68**; **B45**, **51; C6, 15**	DR13, 14; DQ7, 8; DP3, 10
7	A1, –; B57, –; C6, –	DR7, 13; DQ6, 9; DP2, 4	**A2**, **31**; **B50**, **51**; **C**6, **15**	DR7, 7; **DQ2**, 9; DP4, –
8	A1, 33; B65, 57; C6, 8	DR4, 15; DQ6, 8; DP4, 23	**A2**, –; **B39**, **50**; C6, 12	DR11, 13; DQ6, 7; DP2, 4
9	A29, –; B65, –; C3, 8	DR1, 17; DQ2^*^, 5; DP2, 4	A29, **32**; **B44**, **52**; C12, 16	**DR7**, 17; DQ2^*^, –; **DP**2, **11**
10	A25, 33; B62, 65; C3, 8	DR1, 12; DQ5, 7; DP3, 10	**A2**, **3**; B35, **50**; C4, 6	DR7, 17; DQ2, –; DP4, 3
11	A2, –; B35, 49; C4, 7	DR4, 11; DQ7, 8; DP4, –	**A**2, **31**; **B**50, **51**; C6, 15	**DR7**, –; **DQ2**, 9; DP4, –
12	A29, 68; B44, –; C16, –	DR4, 7; DQ2, 8; DP4^†^, 11	A68, 68; **B39**, **50**; C6, 7	**DR8**, **16**; DQ4, 5; **DP3**, **4^†^**
13A			**A**1, **2**; **B44**, 57; **C5**, 7	**DR**7, **12**; DQ7, 9; DP4, 6
	A1, 11; B39, 57; C7, 12	DR4, 7; DQ7, 9; DP3, 4		
13B			**A2^‡^**, **3**; **B44**, **53**; **C4**, **5**	**DR**1, **15**; **DQ5**, **6**; DP2, 6
14A			**A**1, **29**; **B8**, 44; **C7**, **16**	**DR1**, **4**; DQ5, 8; DP4, 4
	A2, –; B44, 51; C5, 15	DR11, 13; DQ5, 7; DP4, 13		
14B			**A29**, **32**; B44, –; **C**5, **16**	**DR7**, **12**; **DQ2**, 7; **DP**4, **11**

*HLA profiles for both recipients and donors. Dash symbol indicates homozygous antigens. Anti-HLA donor antibody specificities (DSA) are displayed in bold type. HLA coincidences between recipient and donor are underlined. 13A and 13B, and 14A and 14B correspond to subsequent vascular transplants in those patients, respectively*.

* Recipient: DQA01*05:01. Donor: DQA01*02:01.

† *Recipient: DPB04:01. Donor: DPB04:02*.

‡ *DSA previously formed after the first vascular transplant*.

The generation of DSA was also confirmed in all patients 1 month after transplantation, with no specific IgG detectable 1 week from the surgery. Subsequent serial analysis showed little variation from the immune profiles obtained at 30 days follow-up, although many antibody titers gradually increase in time until reaching maximum MFI test range at 3 months. On the other hand, the number of donor specific IgG registered and its quantification through MFI did not report specifically higher values in patients with known allograft degeneration. Moreover, high values in both parameters were not necessarily related to aneurysm degradation during the study.

In addition, the CDC crossmatch test showed evidence of complement-fixing antibodies in the serum of all patients. These IgG are capable of activating complement when bonded with the antigen, which may result in cell lysis and could favor allograft rejection. This was confirmed in the antibodies produced not only against donor HLA, but also against cells expressing other alleles that shared epitopes with them. Table 5 shows HLA-typing for the six bone marrow donors used to test complement-fixing ability.

**Table 5:** HLA-Typing for the CDC crossmatch test.

	**HLA-A**	**HLA-B**	**HLA-C**	**HLA-DR**	**HLA-DQ**	**HLA-DP**
1	26, 33	65, 35	4, 8	4, 11	7, 5	3, –
2	2, –	13, 51	2, 6	1, 4	1, 7	3, 5
3	3, 68	61, 53	2, 4	1, 15	1, –	5, 6
4	2, 33	18, 44	5, 7	1, 17	1, 5	2, 5
5	33, 8	44, –	2, 5	1, 15	1, –	5, 6
6	2, 68	70, 53	2, 4	1, 7	1, –	3, 5

*HLA profiles for the six random bone marrow donors used in the Complement-Dependent Cytotoxicity crossmatch test. The numbers represent HLA phenotypes for each subclass. Dash symbol indicates homozygous antigens*.

## Discussion

Vascular transplants represent a unique opportunity to investigate the unmodified immune response to donor antigens in the absence of immunosuppressive medication, unlike solid organ transplantation. At present, it is not an option to systematically treat vascular allograft rejection, as ABO/ Rhesus compatibility has shown no clear benefit (13–15), HLA-matching is generally non-viable owing to the limited stock of vessels, and sustained immunosuppressants are hardly an option in most patients with vascular infection and/ or multiple risk factors and comorbidities (15).

Immunologic behavior of cryopreserved allografts has been the subject of various studies. However, prospective research focusing on the evolution of aneurysmal degenerations and its relationship with histological changes and specific antibody response is scarce. This specificity becomes of paramount importance when trying to establish the immune response triggered by vascular transplantation. Failure to address the issue may partially explain the controversial results published by different authors regarding the antigenicity of cryopreserved arteries and veins, many times reporting opposite conclusions (17–20).

To our knowledge, only three research groups have established the presence of DSA after vascular allograft surgery in humans. Mirelli et al. included seven cryopreserved arteries in 2005 with their last series (21), accounting for 20% of the bypasses, and confirming the presence of DSA with no significant difference in antibody formation between the fresh and the cryopreserved group. Balzer et al. (22) confirmed antibody specificity against donor HLA antigens in 10 fresh venosus allografts, while low dose cyclosporine did not prevent the appearance of DSA. Finally, in 2017 Konrad et al. (23) studied the immune response to 40 fresh and four cryopreserved arteries over a 10-years period. A single blood sample was taken 10–13 months after transplantation, and the results showed the presence of DSA in the absence of random HLA matching in all cases but one.

These studies included a limited number of cryopreserved allografts (11 in total), focusing on fresh vessels, and none reported aneurysmal degenerations that could help to establish an immune profile for failing vascular transplants.

Our research on cryopreserved arteries confirms the production of anti-HLA antibodies in all cases 1 month after implantation when there is no early graft thrombosis. Moreover, we have confirmed the presence of DSA in all 14 patients suitable for specificity study after 1 month, but with no IgG detectable against donor HLA-antigens of the cryopreserved artery explanted and replaced just a few days after the surgery. Human immune system seems to require more than a week of exposure to be able to activate specific antibodies against vascular allografts. This finding was also observed by Balzer et al. (22) in a patient with two sequential fresh venous allografts that failed after 3 and 4 days, respectively, requiring limb amputation. The presence of complement-fixing antibodies also corroborates that cryopreserved arteries can generate an immune response leading to chronic allograft rejection (24, 25).

In light of these immunological results, we suggest, whenever possible, avoiding allograft revascularization in patients with chronic renal failure who fulfill requirements to become potential renal transplant receptors. The aim is to reduce future recipient presensitization that would later prevent from finding suitable donor candidates. This recommendation had previously been made for the use of cryopreserved allografts as haemodialysis access in those patients (26) or as arterial interponates in kidney transplants (27). It is important to remember that 28.5% of our series registered glomerular filtration rates <60 mL/min.

How this immune reaction against specific donor HLA antigens affects the vascular transplant seems more difficult to establish. Unexpectedly, aneurysmal degeneration was not associated with consistent higher values of IgG in comparison with other allografts, while some patients with a high production of IgG did not register aneurysms during follow-up. These results are not easy to interpret and could be related to different levels of immunogenicity for distinctive antigens. Besides which, when multiple cryopreserved arteries are subsequently implanted in the same patient due to degeneration, the time lapse between surgery and the next allograft failure unexpectedly increased in most of the cases, as seen on Table 3. This was observed regardless of progressively higher levels of HLA sensitization and contrasts with consistent data on DSA generation. Taking into consideration the PRA values registered after the first allograft, it would be extremely improbable that no preformed antibodies would be able to recognize alleles from a second vascular transplant. It is noteworthy that we have not found any particular aspect in the immune response of patients with degenerated allografts that makes them stand out from the rest of the subjects in our series. Moreover, cryopreserved arteries from the same donor were implanted into two different recipients (ID 5 and 6 in Table 4), developing aneurysms only in the later. We believe this is the first time that antibody specificity has been studied in patients with cryopreserved artery degeneration, not only by stablishing the DSA generated after the transplant, but also by quantifying its production thereafter.

Focusing on the group of patients with multiple allograft degradations, three out of six cases had a record of two or more native arterial aneurysms. This finding includes both patients with three consecutive degenerations. Although sample size is limited and conclusions should be taken carefully, this percentage of native aneurysmal disease was not observed in the rest of the studied population. Why allografts are more sensitive to degradation in these specific patients is not clear. It reinforces the hypothesis that the failure of cryopreserved arteries is multifactorial and cannot be only related to immune parameters. So far, this is also the first report about aneurysmal degenerations in consecutive cryopreserved arteries within the same patients, again with unexpected results.

The period of time required for aneurysmal degeneration is very variable, ranging from 7 to 71 months in our series. Taking into consideration that many cases did not show previous changes in the diameter of the vessel, suggesting later dilation, we recommend ultrasound controls every 6 months for as long as the bypass remains patent. The aim is to establish early diagnosis and treatment, preventing further complications due to rapid degeneration. We also suggest, whenever possible, avoiding the use of new cryopreserved arteries after a previous episode of allograft degradation, as the evolution toward new aneurysms seems to be a frequent response in these patients.

Degenerated cryopreserved arteries do not show signs of chronic inflammation in the form of mononuclear cell infiltration in half the samples analyzed. On the other hand, C3 and IgG immune complex depositions in the intima and media account for positive immunofluorescence and immunohistochemistry in almost all our series. The results can be interpreted by understanding chronic rejection as an ongoing process affecting the allograft in various consecutive and simultaneous ways. These changes were first described by Plissonnier et al. (28, 29) in their classic publications on an animal model of aortic transplantation, although their timing seems to be subject to important variability, according to the results reported by later authors (30–32). Inflammatory infiltration would directly depend on the phase of chronic allograft rejection, which comprises three differential stages: a first stage of immune recognition initially mediated by the endothelium; a second stage of destruction of smooth muscle cells at the media by antibodies and associated with inflammatory infiltration of the adventitia; finally a third stage of scarring process including intimal proliferation and adventitial fibrosis. After smooth muscle cell antigens disappear, the level of inflammation progressively decreases (33). Our observations suggest that aneurysmal degeneration can take place in all the stages. That could explain the differences reported between samples, especially if we take into consideration that both immunological and non-immunological factors could participate in allograft degradation. Furthermore, the recurrent presence of immune complex deposition denotes ongoing allogenicity despite the alterations suffered by the arterial wall, probably in relation with the remaining donor cellular targets which, even when depleted, never completely disappear.

In particular, we have found very significant the analysis of a degenerated femorodistal bypass formed by two cryopreserved arteries from different donors with end-to-end anastomosis. In contrast to the proximal artery, the sample from the distal allograft shows inflammatory infiltrate and calcification, together with tunica media fibrosis. These findings on two vascular transplants subject to the same haemodynamic forces for identical period of time are probably consistent with a differential response on the bases of disparate immunological compatibility, in a patient already sensitized because of previous revascularization with a cryopreserved artery.

These results support the view that alloimmune reaction takes place in all allografts despite cryopreservation processing, although they cannot verify if this reaction is diminished in any degree. Allaire et al. (34) proved that the immune response is triggered by the cellular components of the allograft and not the extracellular matrix. Cryopreserved allografts present a reduced number of cells (35, 36), particularly endothelial and smooth muscle cells, so a decreased immune response should be expected, with a less severe chronic rejection (30). This could explain the differences observed between fresh and cryopreserved allografts in terms of bypass degeneration, as reported in a systematic review by Fahner and his team (37). The endothelium plays a crucial part in this rejection process, as it is considered one of the most immunostimulatory components in solid organ transplantation (38, 39). Surface endothelial antigens from the donor would participate in chronic vascular injury by sustained stimulation of CD4^+^ T lymphocytes. Consequently, local release of cytokines and growth factors (interleukin-1, tumor necrosis factor, platelet derived growth factor …) would regulate recruitment of inflammatory cells, intimal smooth muscle cell proliferation and accumulation of extracellular matrix (40, 41).

We have also registered a poor performance of infrageniculate allografts in terms of primary, primary assisted or secondary patency, together with an important number of reinterventions. However, these results are in contrast with satisfactory limb salvage and mortality rates. We have to take into consideration that the use of cryopreserved arteries was indicated in challenging situations concerning critical ischemia and native or prosthetic vascular infection, which are associated with an important risk of amputation, morbidity and mortality. Several studies have reported similar outcomes (13, 42), with an emphasis on the dissociation between distal bypass patency and medium to long-term limb and patient survival. This is especially significant, as some authors report that only 45% of patients with critical limb ischemia will remain alive and with both legs 1 year after the diagnosis (43). Moreover, the average candidate for cryopreserved allografts, as described here, is a 72 years old male with multiple cardiovascular risk factors and a history of previously revascularized chronic limb ischemia. These parameters do not help to improve bypass performance and underline the difficulty to treat our patients in the absence of a valid autogenous vein.

The major limitation of this study is the low sample size, which does not allow to draw definite conclusions from our research and lacks statistical power to confirm significant relationships in allograft failure. This problem has been a constant in most publications on human vascular transplants when analysing antibody response or aneurysmal degradation. Also, in order to obtain a sufficient number of degenerated allografts for anatomopathological examination, a second study group had to be established. By including patients already transplanted at the beginning of the research, but whose degeneration took place during the inclusion period, we dramatically reduced the recruiting time, but at the price of creating two separate study groups, both with different aims.

We have also failed to establish immunological or clinical markers able to predict the evolution of cryopreserved arteries once implanted, underlining the importance of a systematic follow-up protocol after vascular transplant surgery. Although this would have been a valuable outcome, we understand that the involvement of both immunological and non-immunological factors in the final failure of vascular transplants would require extensive data collection for longer periods. Larger prospective studies will probably benefit from collaboration between institutions in order to reduce inclusion time and increase sample size. Nevertheless, the paper addresses some interesting and novel aspects of the immune response to cryopreserved allografts and opens the door to future research in the same direction.

## Conclusions

Cryopreserved arterial allografts generate a chronic immune reaction by producing a specific antibody response when HLA mismatching takes place. We have also proved the presence of complement-fixing antibodies, which could favor allograft rejection. Nevertheless, the reaction observed in our research is not necessarily associated with aneurysmal degeneration, showing important variability in terms of presentation time, histopathology, and antibody production. Unfortunately, we have not found specific immune markers that could predict future allograft failure.

Even though allograft performance was relatively poor, patient and limb survival were unexpectedly good for clinical conditions that are usually associated with high morbidity and mortality.

Until new bioengineered tissues become available in future years, rendering present grafts obsolete, cryopreserved arteries remain a valuable option for specific indications, although results are dependent on good candidate selection and systematic follow-up, aiming to prevent the inherent limitations of vascular transplants.

## Data Availability Statement

The raw data supporting the conclusions of this article will be made available by the authors, without undue reservation.

## Ethics Statement

The studies involving human participants were reviewed and approved by Bioethics Committee of the Central University Hospital of Asturias (Oviedo, Spain). The patients/participants provided their written informed consent to participate in this study.

## Author Contributions

MG-G: conception and design, data analysis, and interpretation. RL-M: immunological processing. SB-S and MR-W: clinical follow-up and data collection. MM-H: ultrasound scanning. FÁ-M: statistical analysis. MA-P: vascular surgery supervision. RA-A: immunology supervision. All authors contributed to the article and approved the submitted version.

## Conflict of Interest

The authors declare that the research was conducted in the absence of any commercial or financial relationships that could be construed as a potential conflict of interest.
